# Utilizing Precursor Ion Connectivity of Different Charge States to Improve Peptide and Protein Identification in MS/MS Analysis

**DOI:** 10.1021/acs.analchem.3c03061

**Published:** 2024-01-09

**Authors:** Lily R. Adair, Ian Jones, Rainer Cramer

**Affiliations:** † Department of Chemistry, University of Reading, Whiteknights, Reading RG6 6DX, United Kingdom; ‡ School of Biological Sciences, University of Reading, Whiteknights, Reading RG6 6AJ, United Kingdom

## Abstract

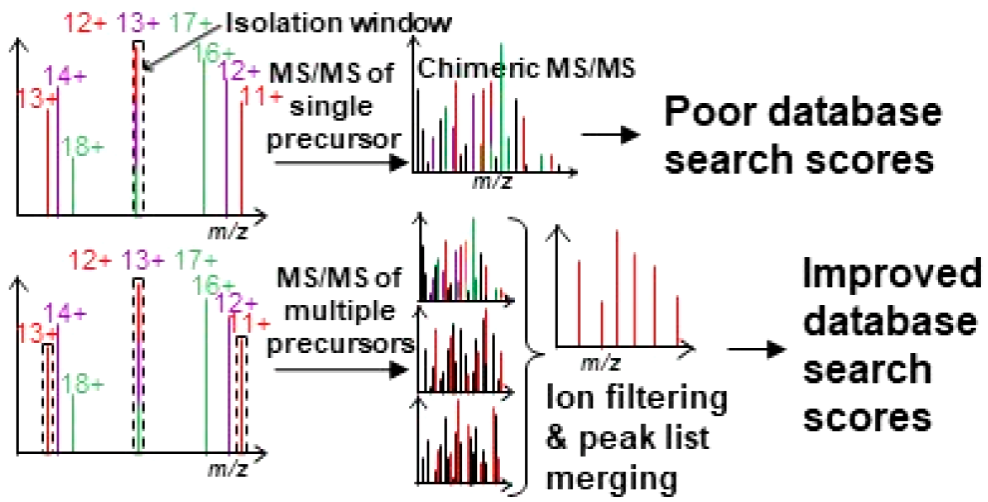

Tandem mass spectrometry (MS/MS) has become a key method for the structural analysis of biomolecules such as peptides and proteins. A pervasive problem in MS/MS analyses, especially for top-down proteomics, is the occurrence of chimeric spectra, when two or more precursor ions are co-isolated and fragmented, thus leading to complex MS/MS spectra that are populated with fragment ions originating from different precursor ions. This type of convoluted data typically results in low sequence database search scores due to the vast number of mixed-source fragment ions, of which only a fraction originates from a specific precursor ion. Herein, we present a novel workflow that deconvolutes the data of chimeric MS/MS spectra, improving the protein search scores and sequence coverages in database searching and thus providing a more confident peptide and protein identification. Previously misidentified proteins or proteins with insignificant search scores can be correctly and significantly identified following the presented data acquisition and analysis workflow with search scores increasing by a factor of 3–4 for smaller precursor ions (peptides) and >6 for larger precursor ions such as intact ubiquitin and cytochrome C.

Tandem mass spectrometry (MS/MS) is widely used for the analysis of intact proteins. In top-down proteomics, it enables the comprehensive analysis of proteoforms and post-translational modifications (PTMs), thus serving a greater understanding of basic biological functions and disease mechanisms as well as the identification of new disease biomarkers.^1^

One of the greatest hurdles experienced in top-down proteomics is overlapping and co-isolated precursor ion signals.^2^ Particularly with electrospray ionization (ESI),^3,4^ or similar ionization techniques that generate multiply charged peptide/protein ions such as LAP-MALDI,^5,6^ the analysis of complex mixtures can be challenging as the diverse range of charge state distributions can result in a mixture of various analyte ions being co-isolated even in relatively tight *m*/*z* windows for MS/MS fragmentation. If up-front separation methods such as liquid chromatography are coupled to MS/MS, some deconvolution can be achieved by exploiting the ions’ temporal (chromatographic) ion signal profile, matching fragment (and precursor) ion intensities with the same temporal profile as is the case in methods like SWATH^7^ or MS^E^.^8^ However, many such strategies are devised for low-charge state, bottom-up proteomics and require the acquisition of (spectral) libraries, i.e., prior knowledge of the peptide’s chromatography and MS behavior, stable chromatographic ion profiles, and/or sophisticated (statistical) analysis tools. In many cases, adequate up-front separation is not even a viable option in top-down proteomics. Thus, these retention-time-based LC-MS(/MS) methods are less effective for the MS/MS analysis of complex proteinaceous mixtures that have not undergone the typical enzymatic digestion path of bottom-up proteomics.

Online and offline separation/fractionation methods have also been utilized simply to decrease the occurrence of chimeric spectra. Commonly used strategies include reducing sample complexity through orthogonal fractionation,^9,10^ the use of ion mobility (IM) to separate analytes online by their collisional cross section,^11,12^ as well as bioinformatic methods that can quantify the effect of chimeric MS/MS spectra on identification and employ a range of deconvolution strategies to deal with complex samples.^13,14^ However, as before, most methods have been optimized to support bottom-up rather than top-down proteomics and require an added dimension of separation/fractionation.

For global bottom-up proteomics, for instance using a yeast data set from the PeptideAtlas repository (https://peptideatlas.org/; PASS00665), it has been estimated that there are on average around 2 precursor ions per MS/MS spectrum. It was reported that for this data set 30% more identifications can be obtained using an iterative search algorithm and attenuating the fragment ions of a previously identified peptide.^15^ The number of precursor ions per MS/MS spectrum obviously depends on the exact analytical workflow and proteome, in particular the separation efficiency prior to MS/MS, precursor ion selection, and the size of the proteome.

For global top-down proteomics, the larger sizes of the precursor ions aggravate the difficulties in separation prior to MS and at the MS and MS/MS stage. Thus, overlapping precursor ions can be as much or even more of an issue than in bottom-up proteomics. Consequently, there have been only a few global top-down proteomics studies. Most of these studies identified only a small number (hundreds) of proteoforms. However, two recent large-scale top-down proteomics studies extended these efforts to ∼20,000–30,000 nonredundant proteoforms, albeit using more than one cell type/line.^16,17^

The use of multiple fragmentation techniques followed by chimeric fragment ion loading has been shown to improve sequence coverage.^18^ However, spectra resulting from chimeric fragment ion loading should not be confused with chimeric MS/MS spectra or their intrinsic occurrence. In general, chimeric MS/MS spectral data with fragment ion information from two or more precursor ions provide unique challenges for the identification of peptides and proteins by conventional database search engines, leading to reduced database search scores, increased false discovery rate, and an erroneously raised number of protein hits due to an increased number of mixed-source fragment ions or weaker fragment ion intensities of the target analyte.^19^ Correctly grouping and assigning fragment ions to one specific precursor analyte and removing nonspecific fragment ions before database searching can inherently improve any probability scoring and are therefore of critical importance.

Here, we present a novel workflow that utilizes precursor ion connectivity by filtering and collating MS/MS data obtained from different charge states but from the same sample to improve protein sequence coverage and database search scores. By using the data of multiple MS/MS analyses of different charge states, filtering each for common fragment ions, and merging them into one peak list, (bio)chemical noise is reduced, and only common protein-specific ions are retained and submitted to database searching. Without applying additional separation and fractionation methods or sophisticated statistical software, substantial improvements in protein identification can thus be made for complex samples in top-down proteomics.

## Experimental Section

### Materials

All MALDI matrix components and analytical standards were purchased from Sigma-Aldrich (Gillingham, UK). HPLC-grade acetone and LC-MS-grade water and acetonitrile were purchased from Fisher Scientific (Loughborough, UK). The dehydrated nutrient agar culture medium was obtained from Oxoid/ThermoFisher (Basingstoke, UK). The *Escherichia coli* (NCTC 13386) bacterial strain was obtained as freeze-dried discs from Pro-Lab Diagnostics (Wirral, UK).

### Sample Preparation

A loopful of *Escherichia coli* (NCTC 13386) stock stored in 70% glycerol was streaked and incubated at 37 °C for 24 h on a solid nutrient agar plate. Approximately 5 μL of biological material was subsequently harvested and resuspended in 1 mL of 1X phosphate-buffered saline (PBS) mixed with 50 μL of 100% trichloroacetic acid (TCA). These samples were then precipitated on ice for 30 min and subsequently centrifuged for 2 min at 13,000 *g*. The resulting pellet was washed once with 500 μL of acetone before being resuspended in 30 μL of 0.1% trifluoroacetic acid (TFA). Following further centrifugation for 2 min at 13,000 *g*, the supernatant was taken and purified using C18 ZipTips (Merck; Gillingham, UK) according to the manufacturer’s instructions. Proteins were eluted in 5 μL of acetonitrile:water (50:50; v/v).

Human adrenocorticotropic hormone fragment 1-17 (ACTH 1-17), human angiotensin II (Ang II), and human bradykinin acetate salt (BK) as well as myoglobin from equine skeletal muscle, ubiquitin (Ub) from bovine erythrocytes, and cytochrome C (CC) from equine heart were prepared at 10 μM in water. A two-peptide mixture (ACTH 1-17 and Ang II); a two-protein mixture of Ub and CC; and a mixture of BK, equine myoglobin, and *E. coli* extract were prepared by using between 0.5 and 5 μL of the above single-analyte solutions and ensuring similar intact analyte ion signal intensities in the mixtures.

### MALDI Matrix Preparation and Sample Spotting

A liquid support matrix (LSM) was prepared by dissolving α-cyano-4-hydroxycinnamic acid (CHCA) in water:acetonitrile (3:7; v/v) to a concentration of 25 mg/mL. After brief sonication, ethylene glycol was added at 70% by volume. The MALDI sample was prepared by spotting 0.5 μL of the LSM onto a stainless-steel MALDI sample plate and adding 0.5 μL of the analyte solution to the LSM.

### LAP-MALDI MS and MS/MS

A detailed description of the in-house developed LAP-MALDI source coupled with a Synapt G2-Si (Waters Corp., Wilmslow, UK) can be found in a previous publication.^20^ For this work, the pulsed beam of a 343 nm laser with a pulse repetition rate of 30 Hz was focused on the middle of the MALDI sample droplets. A 3.0 kV extraction potential with a counter N_2_ gas flow of 150 L/h was applied to the ion transfer tube. Data acquisition was performed within a *m*/*z* range of 100–2000 in sensitivity and positive ion mode. The instrument was manually calibrated over the *m*/*z* range of 100–2000 using cesium iodide and Intellistart software (MassLynx 4.2; Waters). The *m*/*z* value of the target precursor ion was determined, and the quadrupole isolation window was adjusted using low-mass (LM) and high-mass (HM) resolution values to achieve an isolation window with an *m*/*z* width of 2 (1 for the two-peptide mixture) around the *m*/*z* value selected for collision-*in*duced dissociation (CID) MS/MS. Unless stated otherwise, the LM resolution was set to 4.7 and the HM resolution was set to 19. The CID collision voltage was set between 30 and 60 V, dependent on the precursor ion.

### Data Analysis

MS/MS spectra were opened in MASCOT Distiller (Version 2.8.3; Matrix Science, London, UK) for automated peak picking, using a minimum signal-to-noise (S/N) of 5 with baseline correction (isotope distribution with 500 maximum iterations per scan) and a minimum peak *m*/*z* value of 100 and a maximum peak *m*/*z* value of 2000 under “MS Peak Picking” as well as “MS/MS Peak Picking”. The peak list was then exported, containing the monoisotopic masses of the singly charged equivalents of the multiply charged fragment ions detected. Fragment ion signals acquired from each MS/MS analysis across the charge state distribution were grouped within a tolerance of *m*/*z* ± 0.1, providing the mean *m*/*z* value of all grouped ion signals and the sum of the intensities of all grouped ion signals. Each *m*/*z* group’s individual MS/MS intensities were compared between each MS/MS analysis and filtered using a minimum intensity threshold of 100. Unless stated otherwise, after data processing and filtering, only common fragment ions found in the MS/MS analyses of at least 3 different precursor ion windows were selected. This selection criterion provides a practical compromise between an improved filtered fragment ions list using a large number of charge states, thus limiting the number of false positives, and the workflow’s ultimate applicability to complex samples where possibly only 3 charge states can be practically used. The obtained peak list was then searched using MS/MS Ions Search of MASCOT (version 2.7; Matrix Science) against the SwissProt database (version 2021_01; 564277 sequences; 203340877 residues). For MASCOT search parameters “None” was selected for the enzyme, the (precursor) peptide tolerance was set at “10 ppm”, and the MS/MS tolerance was set at “0.2 Da”. The peptide charge was set to “1+”, and “MALDI-QUAD-TOF” was selected as instrument. No fixed nor variable modifications were chosen, and “monoisotopic ions” was selected.

## Results and Discussion

**Figure 1:**
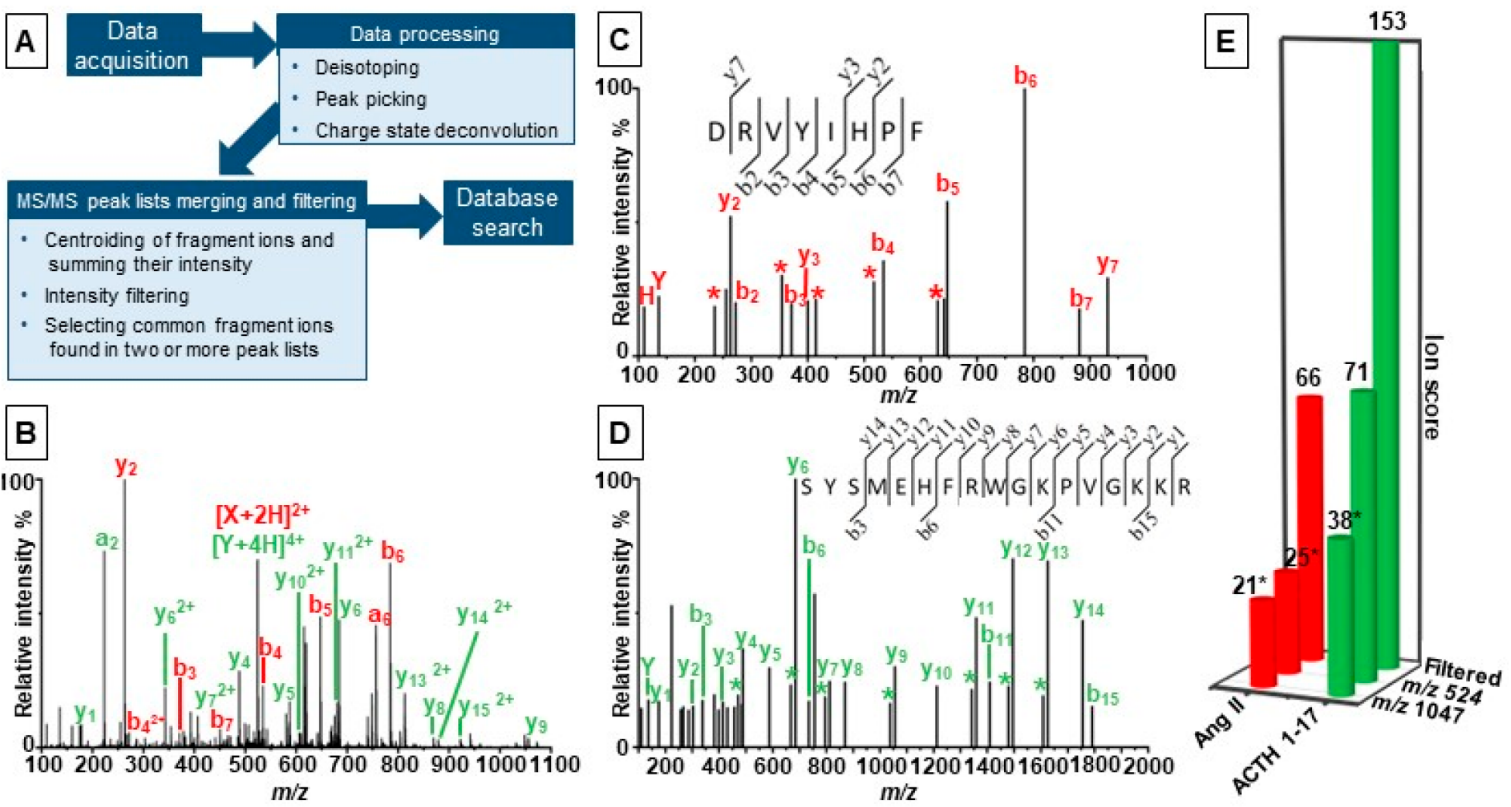
LAP-MALDI MS/MS analysis of a peptide mixture containing ACTH 1-17 (green) and Ang II (red). The workflow for data acquisition, processing, and searching is shown in panel A. Panel B shows the chimeric MS/MS spectrum produced following isolation of the overlapping precursor ions at *m*/*z* 524. Panel C shows the matched fragment ions spectrum following processing, merging, and filtering of the MS/MS spectra for *m*/*z* 349 (Ang II^3+^), *m*/*z* 524 (Ang II^2+^/ACTH 1-17^4+^), and *m*/*z* 1047 (Ang II^1+^/ACTH 1-17^2+^). Panel D shows the matched fragment ions spectrum following processing, merging, and filtering of the MS/MS spectra for *m*/*z* 420 (ACTH 1-17^5+^), *m*/*z* 524 (Ang II^2+^/ACTH 1-17^4+^), *m*/*z* 699 (ACTH 1-17^3+^), and *m*/*z* 1047 (Ang II^1+^/ACTH 1-17^2+^). Panel E compares the protein search scores for the chimeric MS/MS data to the search score for the data from the filtered peak list for each peptide. Scores marked with an * are nonsignificant.

Chimeric MS/MS spectra result from the fragmentation of two or more co-isolated precursor ions. To deconvolute these fragment ions spectra, additional MS/MS data can be obtained from the neighboring ions of the same molecule but different charge state. This strategy utilizes the same sample and MS data, only adding one or more additional MS/MS analyses to the data set. Figure 1A depicts a simple data analysis workflow using this strategy.

To demonstrate proof of concept in the first instance, this strategy and the associated data analysis workflow was tested on a two-peptide mixture containing human ACTH-17 and human Ang II and a two-protein mixture containing bovine Ub and equine CC. These standards were selected on the basis of overlapping isotopic signatures at certain *m*/*z* windows, thus leading to chimeric MS/MS spectra. The overlapping precursor ion species were co-isolated and fragmented by CID. Solely submitting the data obtained from the chimeric MS/MS spectra for database searching led to insignificant search scores due to the presence of nonspecific ions. By adding MS/MS analyses on multiple isolation windows of different charge states and then filtering for only common fragment ions, search scores substantially improved.

For the two-peptide mixture, overlapping precursor ions were observed around *m*/*z* 1047 (theoretical monoisotopic *m*/*z* value of 1047.0467 for doubly protonated ACTH 1-17 and 1046.5418 for singly protonated Ang II) as well as around *m*/*z* 524 (theoretical monoisotopic *m*/*z* value of 524.0270 for quadruply protonated ACTH 1-17 and 523.7745 for doubly protonated Ang II). Figure 1B shows the chimeric MS/MS spectrum for the overlapping precursor ions at *m*/*z* 524.

Further MS/MS analyses of peptide ions that were detected at other charge states and filtering for common fragment ions for each peptide removed (bio)chemical noise and retained only common peptide-specific fragment ions. Figure 1C displays the filtered common fragment ions spectrum based on the MS/MS analyses of the precursor ion windows that included the +1, +2, and +3 charge state of Ang II. For ACTH 1-17, the precursor ion windows of the +2, +3, +4, and +5 charge states were used (see Figure 1D). The LM resolution was set to 4.9, and the HM resolution was set to 15 for isolating the peptides. Fragment ions must be present in three or more isolation windows to be deemed common. Even using a minimum of two isolation windows led to improved search scores.

Following the filtering process, ion scores of database searches using MASCOT substantially increased by a factor of 3–4 with scores of 153 and 66 for ACTH 1-17 (identified as a sequence stretch in P01189) and Ang II (identified as a sequence stretch in P01019), respectively (Figure 1E). Database searches of the peak lists of the chimeric MS/MS spectra led only for the ACTH 1-17 peptide (P01189) to a significant identification using the MS/MS data of the isolation window at *m*/*z* 524. The same data did not result in a significant identification for Ang II. Using the chimeric MS/MS data of the isolation window at *m*/*z* 1047, none of the two peptides were identified with a significant search score (Figure 1E).

Overlapping isotopic patterns and chimeric MS/MS spectra can also be observed for protein mixtures, as shown in Figure S1. Figure S1A displays the MS spectrum of a mixture of bovine Ub and equine CC. In Figure S1B, an enlargement of the *m*/*z* range around the precursor isolation window at approximately *m*/*z* 952 is shown. Precursor isolation was performed with an isolation window width of *m*/*z* 2 to account for the wider isotopologue distributions of proteins; any wider window was at the expense of increasing the number of mixed-source fragment ions, and any narrower window decreased fragment ion detectability. The MS/MS spectrum obtained from the overlapping precursor ions of Ub and CC in this isolation window can be seen in Figure S1C, showing fragment ions matched to both proteins along with many unidentified fragment ions present in this MS/MS spectrum.

Figure S1D displays the filtered common fragment ions spectrum based on the MS/MS analyses of the precursor ion windows that included the +8, +9, +10, and +11 charge state of Ub. For CC, the precursor ion windows of the +12, +13, +14, and +15 charge states were used (see Figure S1E). Fragment ions had to be present in three or more isolation windows to be deemed common.

When the fragment ions peak list was submitted to a database search, bovine Ub was identified with an insignificant ion score of 42 (Figure S1F). Six more MS/MS analyses of the various protein ions with the charge states as labeled in Figure S1A were undertaken. The data of these were subject to the above data analysis workflow with the aforementioned filter thresholds. As can be seen in Figure S1F, the ion scores greatly increased for the merged and filtered fragment ions peak lists compared to those of the chimeric MS/MS data, with all identifications being significant. CC was matched to *Equus caballus* (P00004) with an ion score of 225, and the ion score for Ub was 288, showing an increase by a factor of >6 compared to that of the chimeric MS/MS data.

For the above examples, using two-analyte mixtures prepared with relatively pure standards, isolation windows where only one of the two analytes are isolated, e.g., at *m*/*z* 699 for [ACTH 1-17 + 3H]^3+^ or at *m*/*z* 857 [Ub + 10H]^10+^, can also improve the search scores compared to isolation windows where both analytes are cofragmented. However, using the above strategy by utilizing and filtering for only common ions from at least 3 MS/MS analyses, search scores were still improved. For instance, the ion score obtained for the MS/MS data of [ACTH 1-17 + 3H]^3+^ isolated at *m*/*z* 699 was only 122 compared to 153 for the merged and filtered MS/MS data. Far greater differences were recorded for the protein standards. For Ub, the ion score obtained for the MS/MS data of [Ub + 10H]^10+^ isolated at *m*/*z* 857 was only 146 compared to 288 for the merged and filtered MS/MS data. For some charge states, MS/MS analysis from a single analyte did not result in significant identification. Even for pure standards, the above strategy of merging and filtering several MS/MS analyses from different charge states can reduce analyte-nonspecific background ions and therefore increase search scores.

**Figure 2:**
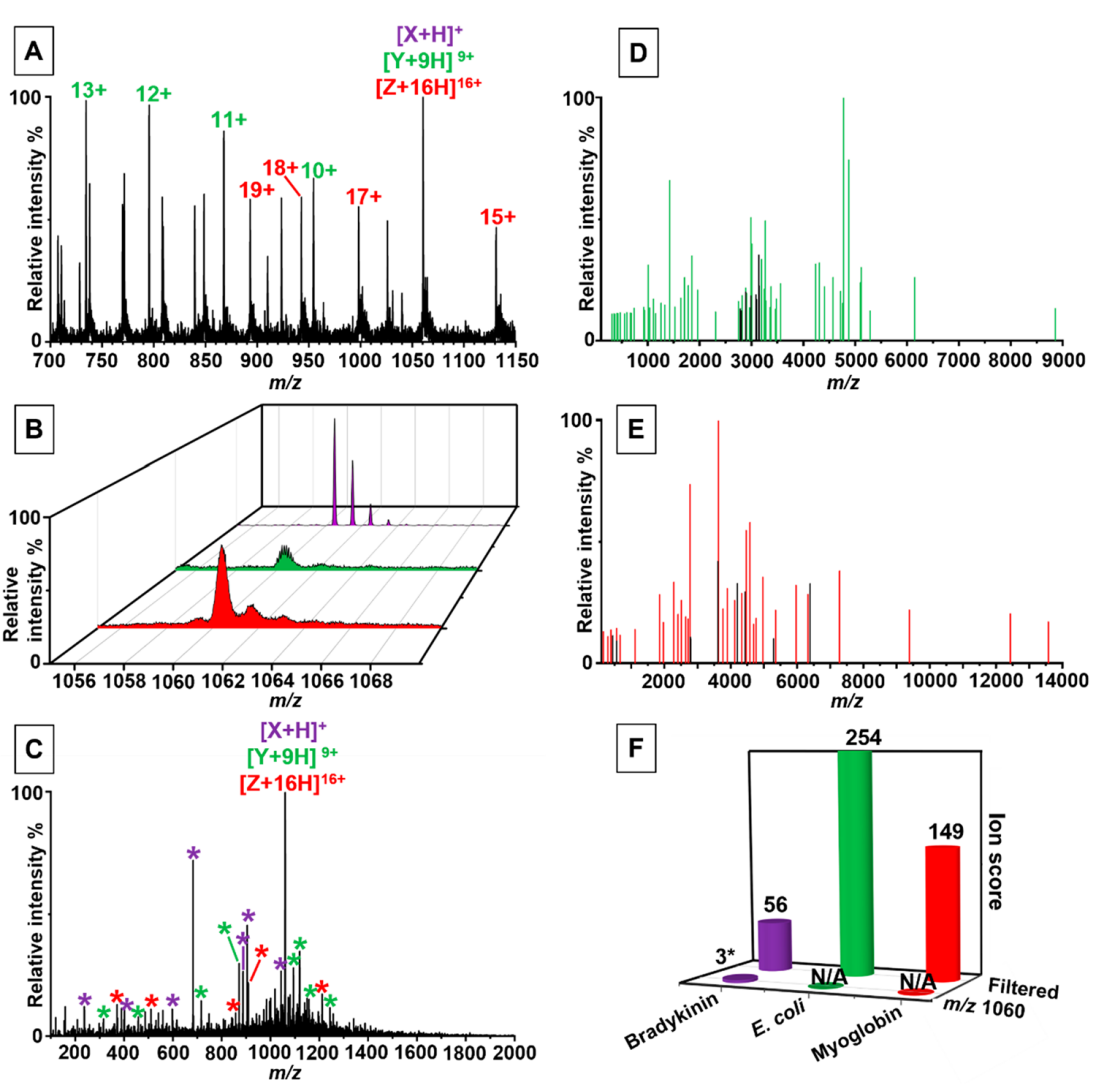
LAP-MALDI MS and MS/MS analysis of a mixture containing human bradykinin (purple), *E. coli* lysate (green), and equine myoglobin (red). Panel A shows the survey mass spectrum with the charge states selected for MS/MS analysis. Panel B displays individual LAP-MALDI MS analyses of each analyte of interest to show their overlapping isotopic pattern. The chimeric fragment ions spectrum in panel C contains fragment ions from both proteins and bradykinin. Panel D shows the matched fragment ions spectrum following processing, merging, and filtering of the MS/MS spectra for *m*/*z* 734, 795, 867, 954, and 1060. Panel E displays the matched fragment ions spectrum following processing, merging, and filtering of the MS/MS spectra for *m*/*z* 893, 942, 998, 1060, and 1130. Panel F compares the protein search scores for the chimeric MS/MS data to search score for the data from the filtered peak list for each protein. N/A denotes that database searching did not return a match for this protein. Scores marked with an * are nonsignificant.

If the sample is more complex and less pure, then greater improvements should be expected. Therefore, this method was also tested with a mixture containing an *E. coli* lysate, equine myoglobin, and human BK, which all have ion signals at approximately *m*/*z* 1060 (Figure 2A/B). The MS/MS spectrum obtained from the fragmentation of all ions isolated at this *m*/*z* value is shown in Figure 2C. The number of fragment ions produced naturally relies on the abundance of each precursor ion being fragmented. If the analyte ion of interest has a relatively low abundance, search scores can be reduced, leading to lower confidence in peptide/protein identification. In this specific case, an insignificant ion score of 3 was obtained for BK (Figure 2E), which was identified as *Ascaphus truei* (P84825). However, the sequence returned by this search is an exact match to human BK. No other matches were returned with the search results.

In total, ten of the most intense peaks were subject to CID MS/MS analysis (all labeled peaks in Figure 2A as well as the peak at *m*/*z* 530). For the BK precursor ion isolation windows (*m*/*z* 530 and 1060), fragment ions had to be present in both of these isolation windows to be deemed common and used for database searching, which then led to an ion score of 56 and a significant match to a sequence stretch in human kininogen (P01042) as well as other BK-containing sequences. Figure 2D displays the filtered common fragment ions spectrum based on the MS/MS analyses of the precursor ion windows that included the +9, +10, +11, +12, and +13 charge state of the unknown *E. coli* protein. For myoglobin, the precursor ion windows of the +15, +16, +17, +18, and +19 charge states were used (see Figure 2E). For these two proteins, fragment ions had to be present in 3 or more isolation windows to be deemed common. Following MS/MS data merging and filtering, myoglobin was correctly identified by the SwissProt entry P68082 with a significant ion score of 149 and the unknown *E. coli* protein was identified as DNA-binding protein HU-alpha (P0ACF0) with a significant ion score of 254 (Figure 2F). For the latter, a single MS/MS analysis isolating the [M+13H]^13+^ only returned an ion score of 55, which is barely above the threshold of 52 for a significant identification, while all other single MS/MS analyses returned lower and insignificant ion scores.

It should be noted that this workflow has been implemented by using a Q-TOF instrument with moderate mass resolving power (up to 10,000 in sensitivity mode). Nonetheless, it was easy to determine the various charge states, even when three substantially different precursor ions were co-isolated and difficult to distinguish as shown in Figure S2. For proteins with higher masses, mass spectrometers with a mass resolving power of >50,000 (FT instruments and multireflection TOFs), particularly in combination with modern deconvolution software such as Mascot Distiller and/or UniDec (http://unidec.chem.ox.ac.uk/), will further support this workflow.

## Conclusions

In this proof-of-concept study, we have developed a workflow to improve sequence coverage and database search scores for complex samples by utilizing multiple isolation windows across the charge state distribution of peptides and proteins, filtering for only common ions and removing ions of insignificance and (bio)chemical noise that are detrimental to peptide and protein identification. The presented data demonstrate the capabilities of the described MS/MS data acquisition and analysis workflow for improving search scores for chimeric spectra. By using the data acquired from multiple isolation windows, filtering for common fragment ions, and merging them into one peak list, our results show substantial improvements in the significance of search scores and an increase in the success rate for protein identifications from (chimeric) MS/MS spectra. Even in instances where complex mixtures can be simplified by fractionation/separation techniques, such as LC and ion mobility, chimeric MS/MS spectra can still occur. The presented workflow will therefore also be highly beneficial in these instances.

## Data Availability

Data supporting the results reported in this paper are openly available from the University of Reading Research Data Archive at 10.17864/1947.000476.

## Supporting Information Available

Additional data figures, including LAP-MALDI MS and MS/MS analysis of a mixture of Ub and CC, and LAP-MALDI mass spectrum of a mixture of BK, myoglobin, and *E. coli* lysate (PDF)

## References

[ref1] Melby J. A.; Roberts D. S.; Larson E. J.; Brown K. A.; Bayne E. F.; Jin S.; Ge Y. Novel Strategies to Address the Challenges in Top-Down Proteomics. J. Am. Soc. Mass Spectrom. 2021, 32, 1278–1294. 10.1021/jasms.1c00099. 33983025 PMC8310706

[ref2] Jeong K.; Babović M.; Gorshkov V.; Kim J.; Jensen O. N.; Kohlbacher O. FLASHIda enables intelligent data acquisition for top–down proteomics to boost proteoform identification counts. Nat. Commun. 2022, 13, 4407 10.1038/s41467-022-31922-z. 35906205 PMC9338294

[ref3] Fenn J. B.; Mann M.; Meng C. K.; Wong S. F.; Whitehouse C. M. Electrospray Ionization for Mass Spectrometry of Large Biomolecules. Science 1989, 246, 64–71. 10.1126/science.2675315. 2675315

[ref4] Fenn J. B. Electrospray Wings for Molecular Elephants (Nobel Lecture). Angew. Chem. Int. Ed. 2003, 42, 3871–3894. 10.1002/anie.200300605. 12949861

[ref5] Cramer R.; Pirkl A.; Hillenkamp F.; Dreisewerd K. Liquid AP-UV-MALDI Enables Stable Ion Yields of Multiply Charged Peptide and Protein Ions for Sensitive Analysis by Mass Spectrometry. Angew. Chem. Int. Ed. 2013, 52, 2364–2367. 10.1002/anie.201208628. PMC3592991 23341077

[ref6] Challen B.; Morris M.; Cramer R. Ultra-High-Throughput and Low-Volume Analysis of Intact Proteins with LAP-MALDI MS. J. Am. Soc. Mass Spectrom. 2023, 34, 991–994. 10.1021/jasms.3c00068. 37102730 PMC10251521

[ref7] Ludwig C.; Gillet L.; Rosenberger G.; Amon S.; Collins B. C.; Aebersold R. Data-independent acquisition-based SWATH-MS for quantitative proteomics: a tutorial. Mol. Syst. Biol. 2018, 14, e8126 10.15252/msb.20178126. 30104418 PMC6088389

[ref8] Silva J. C.; Denny R.; Dorschel C. A.; Gorenstein M.; Kass I. J.; Li G.-Z.; McKenna T.; Nold M. J.; Richardson K.; Young P.; Geromanos S. Quantitative Proteomic Analysis by Accurate Mass Retention Time Pairs. Anal. Chem. 2005, 77, 2187–2200. 10.1021/ac048455k. 15801753

[ref9] Ow S. Y.; Salim M.; Noirel J.; Evans C.; Wright P. C. Minimising iTRAQ ratio compression through understanding LC-MS elution dependence and high-resolution HILIC fractionation. Proteomics 2011, 11, 2341–2346. 10.1002/pmic.201000752. 21548092

[ref10] Villalobos Solis M. I.; Giannone R. J.; Hettich R. L.; Abraham P. E. Exploiting the Dynamic Relationship between Peptide Separation Quality and Peptide Coisolation in a Multiple-Peptide Matches-per-Spectrum Approach Offers a Strategy To Optimize Bottom-Up Proteomics Throughput and Depth. Anal. Chem. 2019, 91, 7273–7279. 10.1021/acs.analchem.9b00819. 31075198

[ref11] Charkow J.; Röst H. L. Trapped Ion Mobility Spectrometry Reduces Spectral Complexity in Mass Spectrometry-Based Proteomics. Anal. Chem. 2021, 93, 16751–16758. 10.1021/acs.analchem.1c01399. 34881875

[ref12] Saba J.; Bonneil E.; Pomiès C.; Eng K.; Thibault P. Enhanced sensitivity in proteomics experiments using FAIMS coupled with a hybrid linear ion trap/Orbitrap mass spectrometer. J. Proteome Res. 2009, 8, 3355–3366. 10.1021/pr801106a. 19469569

[ref13] Stancliffe E.; Schwaiger-Haber M.; Sindelar M.; Patti G. J. DecoID improves identification rates in metabolomics through database-assisted MS/MS deconvolution. Nat. Methods 2021, 18, 779–787. 10.1038/s41592-021-01195-3. 34239103 PMC9302972

[ref14] Wang J.; Bourne P. E.; Bandeira N. MixGF: spectral probabilities for mixture spectra from more than one peptide. Mol. Cell. Proteomics 2014, 13, 3688–3697. 10.1074/mcp.O113.037218. 25225354 PMC4256515

[ref15] Shteynberg D.; Mendoza L.; Hoopmann M. R.; Sun Z.; Schmidt F.; Deutsch E. W.; Moritz R. L. reSpect: Software for Identification of High and Low Abundance Ion Species in Chimeric Tandem Mass Spectra. J. Am. Soc. Mass Spectrom. 2015, 26, 1837–1847. 10.1007/s13361-015-1252-5. 26419769 PMC4750398

[ref16] McCool E. N.; Xu T.; Chen W. R.; Beller N. C.; Nolan S. M.; Hummon A. B.; Liu X. W.; Sun L. L. Deep top-down proteomics revealed significant proteoform-level differences between metastatic and nonmetastatic colorectal cancer cells. Sci. Adv. 2022, 8, eabq6348 10.1126/sciadv.abq6348. 36542699 PMC9770947

[ref17] Melani R. D.; et al. The Blood Proteoform Atlas: A reference map of proteoforms in human hematopoietic cells. Science 2022, 375, 411–418. 10.1126/science.aaz5284. 35084980 PMC9097315

[ref18] Weisbrod C. R.; Anderson L. C.; Greer J. B.; DeHart C. J.; Hendrickson C. L. Increased Single-Spectrum Top-Down Protein Sequence Coverage in Trapping Mass Spectrometers with Chimeric Ion Loading. Anal. Chem. 2020, 92, 12193–12200. 10.1021/acs.analchem.0c01064. 32812743 PMC7845485

[ref19] Houel S.; Abernathy R.; Renganathan K.; Meyer-Arendt K.; Ahn N. G.; Old W. M. Quantifying the Impact of Chimera MS/MS Spectra on Peptide Identification in Large-Scale Proteomics Studies. J. Proteome Res. 2010, 9, 4152–4160. 10.1021/pr1003856. 20578722 PMC3221600

[ref20] Ryumin P.; Brown J.; Morris M.; Cramer R. Investigation and optimization of parameters affecting the multiply charged ion yield in AP-MALDI MS. Methods 2016, 104, 11–20. 10.1016/j.ymeth.2016.01.015. 26827934

