# Supplementary material for: Utilizing Precursor Ion Connectivity of Different Charge States to Improve Peptide and Protein Identification in MS/MS Analysis
Source: Anal Chem. 2024 Jan 9;96(3):985–90. doi: 10.1021/acs.analchem.3c03061 (PMC10809226; doi:10.1021/acs.analchem.3c03061)
Supplement: Supplementary file 1 — ac3c03061_si_001.pdf [file ac3c03061_si_001.pdf]

Supporting Information

Utilising precursor ion connectivity of different charge states to improve peptide and protein identification in MS/MS analysis

*Lily R. Adair,[1] Ian Jones[2], Rainer Cramer[1]**

[1] Department of Chemistry, University of Reading, Whiteknights, Reading RG6 6DX, UK

[2] School of Biological Sciences, University of Reading, Whiteknights, Reading, RG6 6AJ, UK

* R. Cramer
Department of Chemistry
University of Reading
Whiteknights, Reading RG6 6DX, United Kingdom
E-mail: r.k.cramer@reading.ac.uk
Homepage: www.reading.ac.uk/chemistry/about/staff/r-k-cramer.aspx

Table of Contents

Cover Page and Table of Contents **Page S1**

Figure S1: LAP-MALDI MS and MS/MS analysis of a protein mixture containing Ub (red) and CC (green). **Page S2**

Figure S2: LAP-MALDI mass spectrum of a mixture containing human bradykinin, *E. coli* lysate and equine myoglobin. **Page S3**

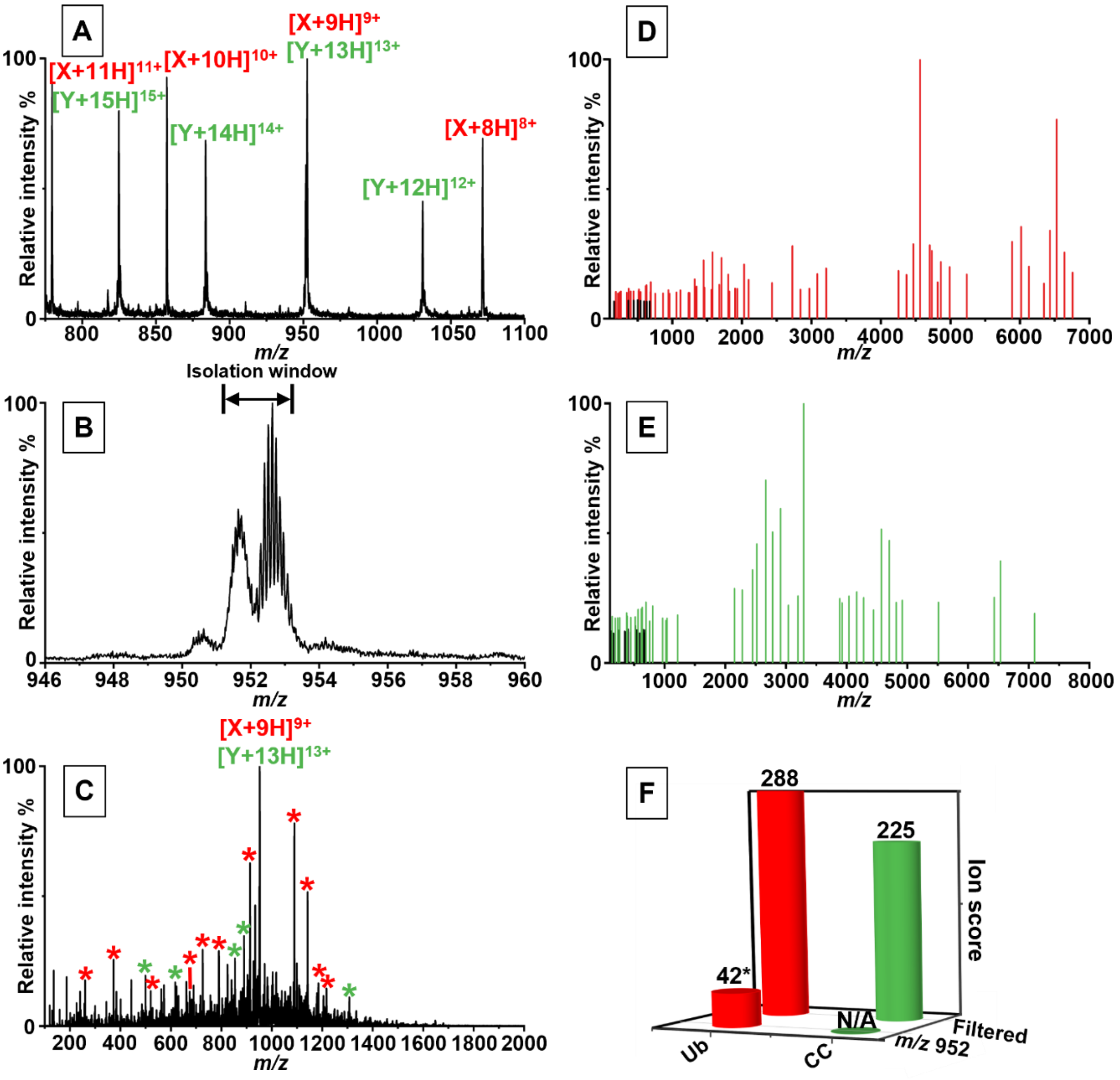

Supplementary Figure S1: LAP-MALDI MS and MS/MS analysis of a protein mixture containing Ub (red) and CC (green). (A) shows the survey mass spectrum with the charge states selected for MS/MS analysis. (B) displays the overlapping precursors at approximately *m/z* 952 prior to isolation and the isolation window with a nominal width of *m/z* 2, allowing both proteins to be transmitted. The chimeric fragment ions spectrum in (C) contains fragment ions from both proteins. (D) shows the matched fragment ions spectrum following processing, merging and filtering of the MS/MS spectra for *m/z* 779 (Ub^{11+}), *m/z* 857 (Ub^{10+}), *m/z* 952 (Ub^{9+}) and *m/z* 1071 (Ub^{8+}). (E) displays the matched fragment ions spectrum following processing, merging and filtering of the MS/MS spectra for *m/z* 825 (CC^{15+}), *m/z* 884 (CC^{14+}), *m/z* 952 (CC^{13+}), and *m/z* 1031 (CC^{12+}). (F) compares the protein search scores for the chimeric MS/MS data to the search score for the data from the filtered peak list for each protein. N/A denotes that database searching did not return a match for this protein. Scores marked with an * are non-significant.

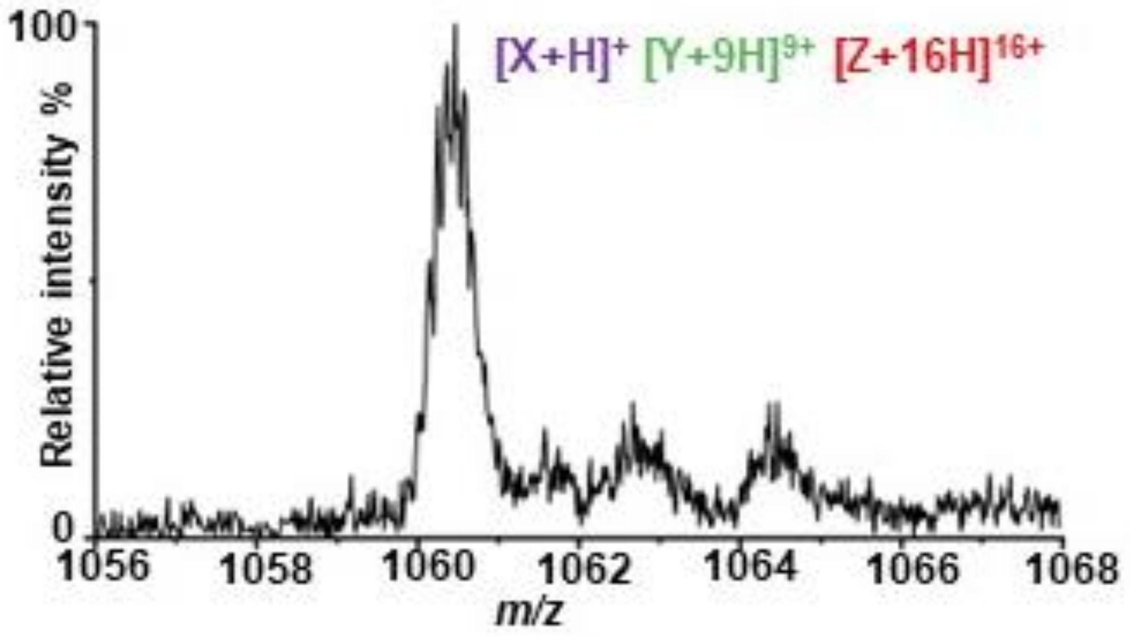

Supplementary Figure S2: LAP-MALDI mass spectrum of a mixture containing human bradykinin, *E. coli* lysate and equine myoglobin, displaying the overlapping precursors at approximately *m/z* 1060 prior to isolation, allowing bradykinin (purple), myoglobin (red) and a proteinaceous analyte from the *E. coli* lysate (green) to be transmitted.
